# Body image and self-esteem under social beauty norms: a qualitative study of the experiences of men and women in Saudi Arabia

**DOI:** 10.3389/fpsyg.2026.1794123

**Published:** 2026-04-08

**Authors:** Ahmed Nasser Alwulaii, Ahmed Mohammed Alasiri

**Affiliations:** Social Studies Department, Sociology, College of Humanities and Social Sciences, King Saud University, Riyadh, Saudi Arabia

**Keywords:** beauty, body image, gender, Saudi Arabia, self-esteem

## Abstract

**Introduction:**

This study examines how social beauty norms shape body image and self-esteem among men and women in Saudi Arabia. While body image has been widely studied in Western contexts, limited research has explored these dynamics in rapidly changing non-Western societies.

**Methods:**

A qualitative approach was employed, using semi-structured interviews with 30 Saudi participants (17 men and 13 women). The interviews explored participants’ perceptions of their bodies and their experiences with social expectations related to appearance.

**Results:**

The findings indicate that beauty standards strongly influence how individuals evaluate their bodies and their social participation. Women reported continuous appearance-based evaluation linked to social acceptance, whereas men framed body expectations more in terms of discipline, fitness, and performance. Participants also described subtle forms of body-related stigma embedded in everyday comments and social interactions, contributing to internalized self-monitoring and feelings of anxiety.

**Discussion:**

The study demonstrates that body image in Saudi society operates within a gendered social framework where the body becomes a site of social evaluation and identity negotiation. It contributes to the literature by providing qualitative insights into body image and beauty norms within the Saudi cultural context.

## Introduction

During a recent encounter with a friend I had not seen for over a decade, I noticed significant changes in how he spoke about his body—as something to be carefully monitored and managed. He carefully tracked his protein intake, carried a water bottle everywhere, and scheduled his workouts with precision. This scene reflects an emerging discourse around the male body that was previously less visible in Saudi society.

The rapid expansion of gyms, the growing number of members, and the availability of 24-h fitness facilities suggest that these practices represent more than simple behavioral changes. Rather, they indicate deeper transformations in the social meaning of the body. The body, particularly the male body, has increasingly become associated with discourses of discipline, productivity, health, and success—discourses reinforced by social media and contemporary visual culture. These dynamics resonate with Foucault’s concept of “docile bodies” (Foucault, 1977), where bodies become subjects of continuous monitoring and regulation. As Bordo (1993) argues, the body functions as a “practical, direct locus of social control,” shaping implicit boundaries between acceptable and unacceptable bodies.

While these transformations are increasingly visible in men’s experiences, an important question emerges: how are women experiencing these changes? In a social context historically characterized by gender segregation, direct access to women’s experiences in public spaces has been limited (Le Renard, 2014). However, the rapid social transformations witnessed in Saudi Arabia in recent years—including greater social openness and evolving practices related to dress and appearance—suggest that the image of the female body is also undergoing renegotiation. For instance, the abaya (a traditional loose outer garment, typically black, that covers the body, worn by women in Saudi Arabia), once primarily associated with concealment and visual modesty, has increasingly become a fashionable and aesthetic garment, sometimes worn without a head covering, particularly in major urban centers such as Riyadh (Al Saud, 2025). These developments raise questions about whether such shifts represent broader societal changes or remain largely confined to specific urban contexts.

Within this evolving context, body image and self-esteem have become central areas of inquiry, particularly given the gendered dimensions of beauty standards (Alwulaii, 2022). Individuals’ narratives about their bodily experiences provide insight not only into their personal relationships with their bodies but also into how societies reproduce norms of acceptance, stigma, and belonging.

Despite extensive research on body image and gender in Western societies (de Casanova and Jafar, 2013; Alwulaii, 2025), studies examining these dynamics in non-Western contexts remain limited. This limitation is particularly evident in conservative societies such as Saudi Arabia, where beauty standards are shaped through complex interactions between culture, religion, and gender norms.

Against this background, this study investigates how men and women in Saudi Arabia describe their bodies and how prevailing beauty standards influence their self-esteem and self-confidence. By focusing on participants’ everyday narratives, the study contributes to emerging scholarship on body image in non-Western contexts and provides new insights into how rapid social change reshapes gendered experiences of the body in contemporary Saudi society.

### Self-esteem and body image

Body image has become a central focus in both psychological research and everyday social experience. Body image plays an important role in shaping self-esteem, as individuals’ evaluations of their bodies influence their overall sense of self-worth.

Body image is commonly defined as the mental representation individuals form of their physical appearance and bodily boundaries (Tylka and Wood-Barcalow, 2015; Pop, 2016; Thompson, 1990). On the other hand, self-esteem refers to an individual’s evaluation or appreciation of their own worth (Robinson and Shaver, 1973). Previous research suggests a strong association between body image and self-esteem, particularly in relation to perceptions of attractiveness, weight, and physical condition (Franzoi and Shields, 1984; Harris et al., 2018; Stowers and Durm, 1996).

Scholars from different disciplines have examined the relationship between body image and self-esteem from multiple perspectives. Psychological research has focused on factors contributing to body dissatisfaction and the psychological outcomes associated with it, such as depression, anxiety, eating disorders, and reduced self-esteem (Overstreet et al., 2010; Merten et al., 2008; Alcaraz-Ibáñez et al., 2021). In contrast, anthropological research has explored the body as a cultural symbol that carries social meanings and representations (Mauss, 2007).

Studies consistently show that negative body image is associated with lower self-esteem, whereas positive body image tends to support higher levels of self-esteem and overall well-being (Aggarwal et al., 2023; Tiggemann and Slater, 2014).

Furthermore, body image is shaped by broader social pressures. Researchers have shown that unrealistic beauty standards promoted through media and social platforms can influence how individuals evaluate their bodies (Fardouly et al., 2015; Fardouly et al., 2017; Aggarwal et al., 2023; Rodgers and Rousseau, 2022). Adopting these standards may create feelings of inadequacy and negative self-evaluation, which can undermine self-esteem (Abuhasabo, 2024). In addition, negative body image and low self-esteem have been linked to personal experiences such as trauma, chronic illness, or bullying (Tylka and Sabik, 2010; Abuhasabo, 2024; Vandenbosch et al., 2022).

### Social beauty norms in a Saudi context

Recent social reforms in Saudi Arabia have had a largely positive impact on women’s rights and have received considerable attention from Saudi scholars (Abuhasabo, 2024; Zaheer et al., 2022). These reforms have influenced several aspects of social and cultural life, including dress practices, mobility, and participation in public spaces. In addition, Western cultural influences have increasingly shaped lifestyle practices among both Saudi women and men, including clothing styles, dietary habits, and leisure activities.

One notable change in recent years is women’s increased participation in the labor market, where they have become important contributors to economic development. However, women and men in Saudi Arabia often navigate these new opportunities while still operating within the boundaries of traditional gender norms (Daya, 2009).

Within this changing social context, beauty standards are also influenced by global cultural flows. Since the early twentieth century, Western beauty ideals have spread beyond their geographical origins. These ideals typically emphasize features such as a thin body, fair skin, blue eyes, and blonde hair (Musaiger, 2015). Such standards have gradually circulated across regions including Asia, the Middle East, and the Arab world.

Beauty standards in Saudi society have developed within a specific cultural and social context shaped by religious values and long-standing social traditions. Traditionally, women’s beauty has been associated with modesty in appearance and adherence to wearing the abaya, reflecting the role of religious and cultural norms in shaping perceptions of femininity and social respectability. Beauty has also been linked to personal grooming and maintaining a socially appropriate image that reflects dignity and respect. In addition, features such as clear skin, long and thick hair, long eyelashes, wide eyes, and fair or smooth skin have historically been regarded as important elements of feminine beauty within the local cultural context (Kashmar et al., 2019).

In earlier periods, particularly several decades ago, a fuller body was also considered a desirable beauty standard and was often associated with health and social well-being (Khalaf et al., 2015). Social occasions such as weddings and family gatherings also play an important role in highlighting beauty norms, as these events provide spaces where appearance, elegance, and personal grooming are displayed and evaluated.

However, with social transformations and increasing exposure to global media in recent decades, beauty standards have increasingly been influenced by global images and ideals circulating through media and social media platforms. These global influences often emphasize thinness, body symmetry, and certain facial features, such as lip shape, which has also contributed to growing interest in cosmetic procedures. Studies have shown that social media exposure has become an important factor influencing body image perceptions among women in Saudi Arabia (AlQahtani et al., 2025). Consequently, beauty standards in Saudi society can be understood as the result of an ongoing interaction between local cultural values and contemporary global influences.

Young people are frequently exposed to these beauty standards through media and popular culture. As a result, striving for an “ideal body” has become increasingly common through practices such as dieting, exercise, or cosmetic procedures. Research suggests that a positive body image can enhance self-confidence and psychological well-being (Hart et al., 2008; Tiggemann, 2014). Individuals may also experience greater social acceptance when their appearance aligns with prevailing beauty norms (Fredrickson and Roberts, 1997; Connell and Messerschmidt, 2005).

### Aim

Saudi Arabia was selected as the context of this study for several reasons. First, the role of women has undergone rapid transformation in recent years (Alwulaii, 2022). Women’s increasing participation in the labor market and leadership positions provides a context in which changes in body image and appearance norms across both genders can be observed. Furthermore, the limited number of studies examining body image and aesthetics in non-Western societies makes this study a valuable contribution to the literature (de Casanova and Jafar, 2013).

Given the relative scarcity of research on body size, self-esteem, and social beauty norms in Arab societies—particularly in the Saudi context—this qualitative study aims to explore how body size influences self-esteem among Saudi individuals within prevailing social beauty norms. The study examines individuals’ everyday experiences in order to understand how beauty standards shape perceptions of the body and personal self-worth. The research is guided by the following questions: How do Saudi individuals describe their bodies in relation to prevailing social expectations? What psychological and social impacts do beauty standards have on self-esteem? What differences exist between male and female experiences?

## Methodology

To address the research questions, this study used a qualitative approach to examine how body image influences self-esteem among Saudi individuals within the context of social beauty norms. Data were collected through interviews that explored participants’ everyday experiences and perceptions of their bodies.

A qualitative approach is particularly appropriate for examining social phenomena that cannot be easily captured through quantitative methods. It enables researchers to explore how individuals interpret their experiences and how social realities are constructed through everyday interactions (Colorafi and Evans, 2016).

### Participants and recruitment

This study received ethical approval from the Ethics Committee at King Saud University (KSU-HE-25-911). All research procedures adhered to the ethical principles outlined in the Declaration of Helsinki (2008 revision). Participants provided digitally recorded informed consent prior to participation.

Participants were Saudi adults living in Saudi Arabia and were required to be at least 18 years old. Recruitment was conducted through multiple channels. First, invitation flyers were posted on widely used social media platforms in Saudi Arabia, including Twitter and WhatsApp. Interested individuals contacted the researchers directly via email using the contact information provided in the recruitment invitation.

In addition, snowball sampling was used to reach further participants. Initial participants were drawn from the researcher’s personal networks, and they were then invited to recommend other potential participants such as friends, relatives, or colleagues who might be interested in taking part in the study. This approach is commonly used in qualitative research when exploring personal or sensitive experiences. A total of 30 Saudi participants took part in the study, including 17 men and 13 women.

### Data collection

Data were collected through semi-structured interviews with participants. The interview guide was developed based on previous research on body image, self-esteem, and social beauty norms. The questions explored participants’ perceptions of their bodies, their experiences with societal beauty expectations, and how these experiences influenced their self-esteem.

Before conducting the main interviews, a pilot interview was carried out to assess the clarity and relevance of the interview questions. Minor adjustments were made to the wording of some questions to improve clarity and flow before the main data collection began.

Participants were encouraged to elaborate on their experiences and provide additional reflections on their bodies, particularly regarding body size and appearance. The interviews were conducted between June and October 2025.

All interviews were conducted via Zoom by the first and the second author, allowing flexibility in scheduling and ensuring consistency in the data collection process, which allowed flexibility in scheduling and ensured consistency in the data collection process. Field notes were taken during the interviews to capture relevant observations, including participants’ tone and non-verbal cues.

Each interview lasted approximately 45–60 min and was audio-recorded and later transcribed for analysis. To ensure confidentiality, each participant was assigned an alphanumeric code. The interview recordings and transcripts were stored securely on an encrypted institutional server.

### Data analysis

The interview transcripts were analyzed using a thematic analysis approach informed by grounded theory principles (Thornberg and Charmaz, 2014). The analysis followed an inductive approach, allowing themes to emerge from participants’ narratives rather than being predetermined.

The analysis began with repeated readings of the interview transcripts to gain familiarity with the data. During this stage, initial open coding was conducted to identify meaningful segments of text related to body image, body size, and self-esteem. These initial codes were then reviewed and compared across interviews.

In the next stage, related codes were grouped into broader conceptual categories. Through an iterative process of comparison and refinement, these categories were organized into overarching themes that captured common patterns in participants’ experiences.

NVivo software was used to assist with organizing, coding, and managing the qualitative data. The themes generated from this process were then used to interpret how Saudi individuals describe their bodies, how beauty standards influence their self-esteem, and how these experiences differ between men and women.

To enhance analytic rigor, the coding process was conducted iteratively, with codes and themes continuously reviewed and refined throughout the analysis. The researchers revisited the transcripts multiple times to ensure that the themes accurately reflected participants’ narratives and that interpretations remained grounded in the data. This process strengthened the credibility and consistency of the analysis (see Tables 1–3).

**Table 1:** Description of the study sample.

*N*	Pseudonym	Gender	BMI
1	Munira	Female	33.7
2	Fahad	Male	37.2
3	Sarah	Female	30.9
4	Noura	Female	26.9
5	Nasser	Male	22.6
6	Khalid	Male	31.6
7	Abdullah	Male	32.2
8	Ibrahim	Male	21.2
9	Kholoud	Female	22.8
10	Tala	Female	22.5
11	Alanoud	Female	22.2
12	Reem	Female	21.5
13	Hessa	Female	22.0
14	Amal	Female	24.2
15	Fatimah	Female	26.5
16	Latifah	Female	22.9
17	Rawan	Female	20.7
18	Abdulaziz	Male	32.4
19	Abdullah	Male	38.7
20	Alwaleed	Male	24.9
21	Rakan	Male	24.0
22	Ahmed	Male	36.8
23	Mohammed	Male	26.9
24	Saad	Male	32.1
25	Hamad	Male	43.2
26	Faisal	Male	25.0
27	Obaid	Male	28.7
28	Sultan	Male	25.1
29	Sabir	Male	27.7
30	Saham	Female	25.6

**Table 2:** Demographic characteristics of participants.

Variable	Category	*N*
Gender	Male	17
	Female	13
Age group	18–24	5
	25–34	11
	35–44	4
	45–54	4
	55–64	6
Employment	Employed	25
	Unemployed	5
Marital status	Single	15
	Married	15

**Table 3:** Overview of themes, codes, keywords, and illustrative quotations.

Main theme	Code	Keywords	Illustrative quotation/Statement
Beauty standards and social pressure	Conditional beauty and appearance-based judgment	Conditional acceptance, appearance, social evaluation	“I feel like people are judging me based on my weight before my personality…‘If you lost a little weight, you’d be gorgeous.’ I felt like my beauty was conditional on it.” (Munira)
Constructing body image and the meaning of the socially acceptable body	Visibility and exclusion in social events	Visibility, exclusion, public image	“The photographer turned the camera away…Even though I danced, I felt like I was behind the scenes.” (Fahd)
	Gendered beauty norms	Gender norms, masculinity, femininity	“For men: an athletic physique, a flat stomach…Social media has made ‘athletic’ the default.” (Fahd,)
	Professionalization of beauty	Workplace appearance, respectability	“At work…appearance is preferred: makeup, colorful abaya…all eyes are on you.” (Tala)
	Body as a condition for social acceptance	Acceptance, belonging, social value	“Sometimes I change my clothes three times…then I go out feeling like I’m not in the mood.” (Sarah)
	Body image as negotiation	Self-acceptance, instability, negotiation	“My relationship with my body is fluctuating…acceptance is not a final state.” (Munira)
	Internalization of external voices	Internal dialogue, guilt, anxiety	“Sometimes I hear the echo of hurtful words in the mirror.” (Sarah)
	Resisting the body as a project	Resistance, body-as-home, wholeness	“My relationship with it is, I would describe it, strange. Some days I feel nice and acceptable, and other days I feel like I do not, and I scrutinize every little detail, like a pimple on my cheek or a small tummy. I also get tired of comparisons—a picture of a girl on Instagram or a casual comment from a friend can ruin my mood…I feel like my body is a house.” (Noura)
Body-related stigma and everyday comments	Functional masculine body image	Functionality, endurance, performance	“The body is not your enemy, you have to learn to work with it.” (Fahd)
	Aging, productivity, and masculinity	Aging body, productivity, decline	“This body served the country…now my steps feel heavy.” (Abdullah)
	Linguistically disguised stigma (WOMEN)	Conditional praise, subtle stigma	“If you lost a little weight, the dress would look better.” (Sarah)
	Achievement reduced to appearance	Devaluation, competence	Effort and success were reduced to a physical evaluation
The body as a site of self-regulation and moral responsibility	Public joking as stigma (men)	Jokes, masculinity, humiliation	“We’ll make you a special door so you can enter…” (Ahmed)
	Managing stigma through silence	Concealment, professionalism	“I laughed so as not to embarrass them.” (Khaled)
	From social pressure to self-surveillance	Self-monitoring, vigilance	“I hear the echo of words in the mirror.” (Sarah)
	Discipline as moral masculinity	Discipline, prestige, respectability	“Any man who neglects his body loses points.” (Nasser)
	Negotiating care versus self-blame	Self-compassion, guilt	“I want change without surgeries or injections that could harm me…sometimes I feel pressure to improve my body, and sometimes I feel like I am not doing enough.” (Khaled)
	Aging and redefining acceptance	Aging, acceptance, resistance	“I try to accept the changes…not demand too much.” (Khuloud)

## Results

The analysis generated four main themes that capture participants’ experiences of body image and self-esteem within the context of social beauty norms in Saudi society. These themes illustrate how beauty standards shape everyday social interactions, influence individuals’ perceptions of their bodies, and affect feelings of acceptance and self-worth. They also highlight important gender differences in how men and women experience social pressure related to appearance. The following sections present these themes and illustrate them through participants’ narratives.

### Beauty standards and social pressure

This theme illustrates how beauty standards function as a powerful social force that shapes individuals’ relationships with their bodies and their self-esteem. Participants described how these standards are reinforced not only through media and social media, but also through everyday interactions within families, workplaces, and social events. The findings also reveal clear gender differences: beauty pressure is more pervasive and persistent in women’s experiences, while among men it is increasingly associated with discipline, prestige, and fitness culture.

#### Beauty standards as everyday “rules” that regulate social behavior

Participants frequently described prevailing beauty standards as unwritten social rules that define what counts as an “acceptable body” and appearance. For women, these standards often emphasize thinness, clear skin, well-groomed hair, and elegant clothing. For men, they tend to focus on an athletic body, a flat stomach, and a respectable style. In this sense, appearance becomes a criterion through which individuals feel socially evaluated.

Munira described how these standards shape how others judge her:

“I feel like people are judging me based on my weight before my personality. At a family gathering someone said, ‘If you lost a little weight, you’d be gorgeous.’ The comment sounded nice, but it hurt. I felt like my beauty was conditional on it.”

Similarly, Fahd reflected on how these expectations appear in social situations:

“At my cousin’s wedding, the photographer was choosing who to film while they were performing the traditional dance. When I got close, he turned the camera away. Maybe it wasn’t intentional… but the message I received was that I didn't fit the scene.”

Fahd also emphasized how social media reinforces these standards:

“For men, it seems necessary to have an athletic physique and a flat stomach. Social media has made the ‘athletic body’ the default image.” These testimonies demonstrate that standards don’t operate through “formal texts”, but rather through social expectations that make the body a constant subject of measurement and comparison. Controlling one’s appearance becomes part of daily “social conformity.”

These accounts illustrate how beauty standards operate through everyday social expectations rather than formal rules. Participants described feeling constantly evaluated through their appearance, which encourages continuous comparison and self-monitoring. In this way, regulating one’s body and appearance becomes part of everyday social conformity.

#### Beauty pressure in the experiences of women and men

Many sources, from stories to everyday events, reveal the pressures men and women face regarding beauty. Women, in particular, have shared numerous experiences about beauty pressure, its sources, and how it affects them. For example, many women mentioned that the sources of pressure are not singular, but rather numerous and varied. Pressure does not come from a single source, but from several sources within society, such as family, relatives, friends at social events, colleagues, or even through the use of filters on social media. For instance,

Sarah explained how such pressure can influence her behavior:

“My husband sometimes compares me to a TV presenter, an actress, or even a social media celebrity. Sometimes I change my clothes or look for a dress that doesn’t accentuate certain flaws or features of my body. Sometimes I change my clothes three times, then I go out feeling like I'm not in the mood.”

In another example, Tala described how beauty standards aren’t limited to social occasions, but have shifted from the social sphere to the professional sphere.

“At work…an attractive appearance is preferred: a colorful abaya, full makeup, even nail polish on the hands. Some shops even require nail polish for employment, and a presentable physique is also necessary. At my job, all eyes are on you…not to mention the physical contact with men…and their comments.”

Here we see that the pressure on women is not just about weight, but extends to minute details (clothing, skin, hair, hands), making the standards more complex and more costly, both psychologically and financially.

“The pressure comes from simple comments. Even when I’m silent, I feel like I’m being judged by looks.”

These narratives demonstrate that beauty pressure for women often extends beyond body weight to include detailed expectations related to clothing, skin, and overall presentation. This makes beauty standards more complex and emotionally demanding. Participants described feeling that their appearance is constantly evaluated, which encourages continuous self-monitoring and reinforces the social regulation of the body.

### The body as a condition for social acceptance

This theme shows how the body functions as a condition for social acceptance in participants’ narratives. For many women, the body is not perceived merely as a personal attribute, but as a factor that influences social acceptance in family settings, social gatherings, and sometimes even in professional contexts. In this sense, women described the body as a site through which their worth and respectability are evaluated.

Participants’ accounts suggest that aesthetic pressure is experienced differently by women and men. Women described the body as closely connected to social acceptance, where appearance becomes a key factor in how they are perceived within their social environment. In contrast, men described beauty pressure as less pervasive, but increasingly present through social media ideals, jokes among friends, and expectations related to prestige and self-discipline.

For example, Fahad talks about pressure through jokes and appearance:

“Sometimes the pressure comes from jokes. During Eid, I had just left the mosque after praying and was going to my family home to greet my grandparents. One of my relatives said, ‘What do you think about taking a picture of all of us?’ While they were taking the picture, the one taking it said, ‘Fahad, we need to take a picture of you separately, because the photo will barely fit your whole body.’ They all laughed, and I laughed too.”

Nasser, however, represents a different model: he sees pressure as potentially transforming into a moral standard. He says,

“Discipline is the foundation of my self-respect…I feel that any man who neglects his body, like a belly or a large chest, loses his prestige.”

This type of discourse reveals that some men deal with beauty standards by “rebranding” them as standards of health and discipline, which gives them greater legitimacy and reduces the recognition that they are social pressures.

These narratives illustrate how body standards can operate as a form of social evaluation. For some men, appearance-related expectations are reframed as values of discipline, health, and responsibility, which makes them appear legitimate rather than socially imposed pressures. At the same time, the findings indicate that women experience more persistent forms of aesthetic pressure linked to social acceptance and appearance. Overall, these experiences demonstrate how beauty standards function as a subtle mechanism of social control that shapes identity, behavior, and self-esteem.

### Constructing body image and the meaning of the socially acceptable body

The findings here reveal how participants construct their perceptions of their bodies and the social meanings associated with what constitutes an “acceptable body” within the Saudi social context. Participants’ narratives demonstrate that body image is not simply a direct reflection of physical form, but rather a fluid social construct shaped through interaction with prevailing social expectations and gender norms. Participants described body image as a dynamic process shaped by social interactions and personal reflections. Women often described their bodies as sites of continuous negotiation between self-acceptance and social expectations. In contrast, men tended to frame their bodies more in terms of function and physical capability rather than aesthetic appearance.

#### Constructing body image in women’s experiences

Women’s narratives show that body image is constructed through an ongoing negotiation between the self and societal norms. The body is not viewed as a static or neutral entity, but rather as a space of daily conflict between attempts at self-acceptance and social pressure. This is clearly reflected in the language used by the participants, who employed metaphors such as home, journey, and reconciliation to describe their relationship with their bodies.

Munira describes her relationship with her body, saying:

“My relationship with it is fluctuating…Sometimes I say, ‘This is my body that carries me, and I have to take care of it…Even if you’re accepting it, you have to accept it…Despite the negativity you face from criticism and other things…you prefer to accept it, especially when the criticism around you subsides.”

This description reflects an awareness that acceptance is not a final state, but rather a long process that requires continuous psychological effort.

Similarly, Sarah points to this internal tension when she says:

“Your body is what carries you, and it’s where you feel pain or other emotions…But accepting it feels difficult to achieve…Whether you lose weight or gain weight, you always feel anxious…There are extra things here that you have to get rid of…If you eat, you feel guilty, afraid of gaining weight, and sometimes I hear the echo of hurtful words in the mirror.”

This indicates that a woman’s view of her body is directly influenced by external voices and social comments that are internalized and transformed into an internal dialogue.

Noura demonstrates a conscious attempt to resist turning her body into a “perpetual improvement project,” saying:

“My relationship with it is, I would describe it, strange. Some days I feel nice and acceptable, and other days I feel like I don’t, and I scrutinize every little detail, like a pimple on my cheek or a small tummy. I also get tired of comparisons—a picture of a girl on Instagram or a casual comment from a friend can ruin my mood…I feel like my body is a house.”

This discourse reflects an implicit rejection of beauty standards that demand women constantly improve, and points to a quest to redefine the body as a living space, not an instrument of evaluation. Furthermore, the metaphor of the body as a “house” suggests a shift from viewing the body as an object of evaluation to understanding it as a personal space of dwelling, safety, and lived experience. It reflects an attempt to reclaim the body as a place of comfort and identity rather than a project of continuous improvement.

Overall, these narratives reveal that women perceive their bodies as constantly visible and socially evaluated, and that their body image is shaped by a constant awareness of how others perceive them, making the body inextricably linked to social value and acceptance.

#### Constructing body image in men’s experiences

In contrast, men’s narratives reveal a different perception of body image, viewing the body more as a tool for performance and function than as an aesthetic object. Men focus on the body’s capacity for movement, work, endurance, and fulfilling socially expected roles, rather than on its physical appearance.

Fahd describes this perception, saying his relationship with his body is like that of a friend:

“I get annoyed by the stairs, I get tired in the heat, my body smells bad after walking in the open air, and the heat here is unbearable…I only feel comfortable and light when I walk for 20–30 mins. I think it’s something psychological…The body isn’t your enemy, but you have to learn to work with it.”

This description reflects a functional relationship based on cooperation with the body to maintain daily functioning. As for Abdullah, he connects his body to his work and service history, saying:

“This body served the country and stood in the sun and dust—now I’m bothered by the heaviness of my steps and shortness of breath. My stomach and belly have gotten big. It’s difficult to balance the desire to eat with maintaining my body…and sometimes I feel like I’m being self-critical, especially when I eat a traditional dish” (a popular dish served at feasts and celebrations in Saudi Arabia.)”

Here, physical change is interpreted as regression. In terms of competence and capability, there is no loss of attractiveness or social acceptance. This reflects a common masculine perception where the body is evaluated through performance and productivity, especially in later life. Generally, men’s narratives do not exhibit the same aesthetic anxieties as women’s; rather, the focus is on physical capability and its symbolic representation of masculinity, responsibility, and independence. This is reflected in men’s reluctance to mention the importance of beauty, which may represent a subtle, unspoken pressure due to gender considerations.

Overall, the findings indicate that the construction of body image follows a clear gendered logic. Women are more frequently evaluated through appearance and social expectations related to beauty, whereas men are more often evaluated through performance and functionality. Despite these differences, both genders recognize that the body is not purely personal but embedded within social meanings and cultural expectations that shape how individuals understand and relate to their bodies.

### Body-related stigma and everyday comments

This theme illustrates how body-related stigma appears in everyday social interactions through direct and indirect comments. Participants described how remarks about the body often occur in contexts that appear ordinary or harmless, such as the workplace, school, or social gatherings. However, these comments are not experienced as isolated events; rather, they form a recurring pattern of interaction that reproduces standards of beauty and social acceptance.

Participants frequently described body-related comments as subtle mechanisms through which appearance becomes a legitimate topic of social evaluation. These comments are often framed as humor, advice, or casual observations, which makes them difficult to challenge. Over time, such interactions contribute to shaping how individuals perceive their bodies and their social value.

#### Stigma in women’s experiences

Women’s accounts indicate that body-related stigma often appears in the form of indirect comments presented as advice or conditional compliments. Although these remarks may seem positive or well-intentioned, they often carry implicit judgments about the body and its conformity to social beauty standards.

Sarah described how such comments can transform a positive moment into an evaluation of appearance:

“She told me, ‘If you lost a little weight, the dress would look better… Some comments just feel unnecessary…I feel it would have been better if she had kept quiet.”

Similarly, Noura described how such remarks can undermine confidence even when they are presented politely:

“Sometimes people say things that sound like advice, but they make you feel like your body is the most important thing about you.”

This clearly demonstrates how a successful experience or personal effort was reduced to a physical evaluation, as if physical appearance were the ultimate standard of judgment. Similarly, Noura describes a similar experience, revealing the profound emotional impact of these comments, especially when they come in a context that is supposed to be appreciation of effort or performance.

These narratives illustrate how body-related stigma toward women often operates through subtle and socially acceptable language. Because the comments are framed as advice or praise, they are more difficult to confront and are often internalized rather than openly challenged. As a result, women may experience a persistent sense that their appearance is being constantly evaluated, which can undermine feelings of competence and self-worth.

#### Stigma in men’s experiences

In contrast, men describe body-related stigma as more direct and sarcastic, often appearing as a public joke or a quick remark expected to be accepted without showing discomfort. However, accounts reveal that this form of stigma is not without psychological impact.

Ahmed says:

“I remember in my meetings with colleagues during breaks, one of them would say, ‘We’ll make you a special door so you can enter…’ and another would say, ‘Go to a tent tailor to have a thobe made for you…’”

This is a clear example of the tension between expected social performance (laughter and participation) and internal feelings (hurt and discomfort). Similarly, Khaled describes a situation in an educational context:

“One of my students is very overweight. He said, ‘Teacher, the chair is too small…’ Another student said, ‘Teacher, I have a chair…’ I laughed when I saw the student was a student so as not to embarrass them.”

These accounts reveal the tension between expected social behavior and personal emotional responses. Men often feel compelled to respond with humor in order to maintain their social role or masculine image, even when the comments are uncomfortable. Unlike women’s experiences, stigmatization among men tends to appear as momentary humor rather than sustained evaluation. However, repeated exposure to such remarks can still generate embarrassment and influence social participation.

Overall, the findings indicate that body-related stigma operates through different gendered mechanisms. For women, stigma often takes the form of subtle and persistent comments that reduce identity and achievement to physical appearance. For men, stigma more frequently appears through humor and sarcasm that are socially expected to be tolerated. Despite these differences, participants emphasized that the body becomes a public object open to commentary and evaluation. Through everyday interactions, these remarks reinforce beauty standards and contribute to shaping body image and self-esteem by creating a sense of constant observation and judgment.

### The body as a site of self-regulation and moral responsibility

The findings here reveal that, in the participants’ experiences, the body is understood not merely as a biological entity or external appearance, but as a central site of self-regulation and moral responsibility. Prevailing beauty standards function not only as external, socially imposed rules, but are also internalized and reproduced, transforming the body into a space where self-judgment is exercised and where discipline, worthiness, and social value are measured.

Participants’ accounts show that individuals do not simply respond to the comments or gazes of others, but develop subjective patterns of continuous monitoring and evaluation of their bodies, holding themselves accountable for conforming to or failing to meet these standards. The findings also reveal clear gender differences in the nature of this responsibility and the discourse through which it is framed. For women, it takes on a moral dimension linked to social acceptance and femininity, while for men, it is associated with discipline, merit, and prestige.

#### From social pressure to self-surveillance

Data shows that the influence of beauty standards extends beyond direct social interaction, becoming part of an individual’s internal dialogue. Many participants describe a state of constant vigilance regarding their bodies, where past comments or social media images become internal voices that continuously monitor and evaluate them.

Sarah described this experience by explaining that she sometimes:

“Hears the echo of words in the mirror,”

Similarly, Noura explained how social media images can influence how she feels about her body:

“Sometimes a single picture on Instagram can change my mood.”

These narratives illustrate how reflecting the shift in the power of evaluation from external to internal. This shift from social control to self-control makes the body a constant project of review and improvement, placing the individual in a perpetual state of self-evaluation.

This self-monitoring appears more intense and persistent among women, who are expected to pay constant attention to minute details related to appearance, such as weight, skin, and clothing. Among men, self-monitoring appears more selective, often linked to indicators of physical performance or fitness rather than detailed physical appearance. However, both genders share the understanding that the body is no longer a neutral space, but rather an internal domain where social power is indirectly exercised.

#### Bodily discipline as a gendered moral value

The narratives of the participants, particularly the men, reveal a clear discourse linking the body to moral discipline and personal worthiness. In this context, attention to the body is seen not only as a healthy choice, but also as evidence of seriousness, responsibility, and even social entitlement. Nasser expresses this perception when he says, “Any man who neglects his body loses points in terms of impression,” a statement that reveals the transformation of the body into a moral standard for judging oneself and others.

This discourse redefines modern masculinity as being tied to controlling and regulating the body, and it imbues beauty standards with a moral character that lends them greater legitimacy. Body discipline is presented here as a positive value, while neglecting one’s body is reframed as a moral failing, not simply a physical difference or a health condition.

In contrast, body discipline appears differently in women’s narratives. Women are expected to care for their bodies not as a sign of seriousness or discipline, but as a prerequisite for social acceptance and “appropriate” femininity. Tala (29 years old) describes how her body becomes part of her professional evaluation, adding the further moral burden of projecting a socially “acceptable” image in the public sphere. Thus, while men are socially rewarded for body discipline as a sign of strength, women are held accountable for any deviation from these norms as a failure to fulfill their social role.

#### Negotiating care versus self-blame

The results show that participants are engaged in a continuous negotiation between two opposing discourses: one of body care and self-compassion, and the other of blame and rigid responsibility. This tension is evident in the narratives of participants who are trying to improve their relationship with their bodies without falling into the trap of cruelty or obsession.

Khaled expresses this struggle when he refers to his desire for “change in my body without surgeries or slimming injections that could cause me serious health problems,” a statement that reflects a conscious attempt to redefine bodily responsibility in a less harsh way. In contrast, Nasser describes harsh responses to any weight gain, manifested as self-flagellation and the imposition of strict regimes, revealing the high psychological cost of turning the body into a moral project.

For women, this negotiation takes on an additional dimension related to age and aesthetics. Khuloud expresses her desire to “try to accept the changes that happen to my body. I’m still young, and age dictates that one should live their life as they choose and not demand too much,” attempting to redefine body care without complete submission to standards of perpetual youth. These narratives reveal that body care is not a neutral practice, but rather a socially charged and morally charged process, where the desire for acceptance intersects with the need to maintain psychological well-being.

Overall, the findings indicate that beauty standards in Saudi society not only shape body image and self-esteem but also transform the body into a site of self-regulation and moral responsibility. While women experience these expectations primarily through appearance and femininity norms, men more often interpret them through discourses of discipline and prestige. In both cases, the body becomes a socially regulated domain through which individuals evaluate their worth and social standing.

## Discussion

This study examined how societal beauty standards in Saudi Arabia shape individuals’ perceptions of their bodies and their sense of self-esteem. The findings highlight significant gender differences in how body image is experienced and negotiated within everyday social contexts. Therefore, we sought to answer specific questions that guided the investigation: how Saudi individuals describe their bodies in light of social expectations, what are the psychological and social impact of beauty standards on self-esteem, and what are the differences in experience between males and females.

The findings suggest that participants understand the body not simply as a biological characteristic but as a socially evaluated entity shaped through ongoing interaction with others. This interpretation aligns with sociological and feminist perspectives that conceptualize body image as socially constructed within gendered cultural contexts rather than as a direct reflection of physical appearance (Bordo, 1993; Bartky, 2015).

Participants’ narratives indicate that women experience the body as a visible and continuously evaluated social space. This reflects broader gendered expectations in which women’s social value is closely tied to appearance. Similar patterns have been documented in body image research showing that women are subject to stricter aesthetic scrutiny and pressure to conform to narrow beauty standards (Wolf, 1998; Fredrickson and Roberts, 1997).

However, this study adds to this literature by illustrating how these dynamics operate within the Saudi context across everyday settings such as family gatherings, workplaces, and the culture of social photography. In these contexts, the body becomes an implicit condition for social acceptance and belonging, which may lead some women to avoid photography or symbolically withdraw from social situations. While this pattern was most clearly illustrated in men’s narratives, similar tendencies may also be present among women, although they were less explicitly expressed in the data.

In contrast, men’s narratives suggest a different social framing of the body, where it is understood primarily in terms of performance, endurance, and functional competence rather than aesthetic appearance. This pattern has been observed in studies of masculinity and the body, where bodily anxiety is reframed within a discourse of health, discipline, and competence rather than explicit beauty (Connell and Messerschmidt, 2005; Gough, 2007).

These findings are consistent with the literature indicating that men are less inclined to express aesthetic anxiety in direct language, despite their increasing adherence to standards of fitness and physical discipline with the rise of fitness culture and image-driven social media (Pope et al., 2000).

The results therefore suggest that body image in Saudi society operates within a gendered framework. Women tend to be socially evaluated primarily through appearance and aesthetic conformity, whereas men are more often evaluated through performance, endurance, and bodily discipline. Although these pressures are expressed differently, participants’ narratives indicate a shared understanding that the body is not purely an individual matter but a social entity shaped by expectations and cultural meanings.

The findings suggest that beauty standards influence not only individuals’ perceptions of their bodies but also their self-esteem and patterns of social interaction. Many participants expressed feelings of anxiety, guilt, and dissatisfaction—emotions documented in numerous psychological and sociological studies that have linked body dissatisfaction to low self-esteem, particularly in contexts where social comparisons are intensified (Cash et al., 2002; Fardouly et al., 2015).

Participants’ accounts—especially those of women—also indicate that social comments and media images often become internalized as persistent self-evaluations. This reflects a shift from external social monitoring to internal self-surveillance. Previous body image research has similarly described this process as the internalization of dominant beauty standards, through which individuals become increasingly self-monitoring (Smolak and Thompson, 2009; Tylka and Wood-Barcalow, 2015).

Previous research has shown that this pattern of self-monitoring is associated with increased body anxiety, eating disorders, and decreased self-efficacy (Merten et al., 2008). The findings of this study further suggest that individuals not only respond to these pressures psychologically but also adjust their social behavior accordingly. Some women described strategies such as avoiding photography or limiting their presence in social gatherings. Similar behaviors have been documented in qualitative research as forms of “preventive withdrawal” aimed at reducing exposure to stigma (Pausé, 2012; Puhl and Heuer, 2009). Men, however, do not tend to withdraw completely. Instead, they appeared to manage their presence in social situations by adjusting their location and movement, a pattern that may be inferred from participants’ accounts, where they described subtly adapting their behavior—for example, by laughing off stigmatizing remarks or remaining physically present while minimizing attention to their bodies. This pattern is less frequently discussed in the literature but may indicate a silent psychological cost associated with maintaining an acceptable masculine image.

Participants’ strategies—such as avoiding photography, limiting their participation in social gatherings, or laughing off comments about their non-conforming body image—can be understood as everyday practices of managing stigma. Rather than withdrawing completely from social spaces, individuals attempt to cope with others’ gazes and comments by adjusting their presence and controlling what is revealed about their bodies. In this sense, participants negotiate what may be described as a “partial” or vulnerable social identity, which requires continuous impression management in order to reduce the risk of demeaning or stigmatizing situations. This interpretation resonates with Irving Goffman’s analysis of stigma as a social process through which individuals manage potentially discrediting attributes in everyday interactions (Goffman, 2009).

These findings highlight that the impact of beauty standards on self-esteem extends beyond individuals’ internal feelings. They also reshape patterns of social participation, individuals’ presence in everyday settings, and their sense of comfort and security in social spaces. In this way, self-esteem appears not merely as an individual psychological trait but as the outcome of a complex interplay between psychological experiences and broader social expectations.

An important contribution of this study is the identification of subtle forms of body stigma embedded in everyday interactions. Participants frequently described comments framed as “advice,” humor, or conditional praise. Although these remarks often appear harmless, they function as mechanisms of social regulation by reminding individuals of expected bodily norms. Similar dynamics have been documented in research on everyday stigma, where seemingly casual remarks reproduce social hierarchies and reinforce conformity to dominant body ideals (Cash and Pruzinsky, 2002; Calogero et al., 2011). Participants’ accounts also illustrate how personal achievements or efforts may be reduced to physical evaluation, reinforcing the association between women’s social worth and their physical appearance. In contrast, men often encounter more direct forms of stigma, such as public jokes or sarcasm, which they are expected to tolerate without expressing discomfort. Research on masculinity suggests that such stigma is frequently managed through silence or forced laughter in order to maintain a coherent masculine identity (Smolak and Thompson, 2009; Courtenay, 2000). Although this form of stigma may appear less severe on the surface, its psychological consequences can be significant, particularly when these situations are repeated and begin to shape individuals’ social behavior or sense of embarrassment. The findings also suggest that, for some men, body discipline becomes reframed as a moral standard associated with prestige, respect, and social worthiness. This interpretation resonates with previous analyses linking bodily discipline to moral responsibility in neoliberal contexts, where body management is framed as a measure of individual merit (Rose, 1999; Crawford, 1980).

In contrast, women bear a different moral burden, namely the expectation of aesthetic conformity as a condition for social acceptance and “appropriate” femininity. These findings suggest that gender differences lie not only in the intensity of stigma but also in the ways it is expressed and managed. Women appear to experience a more cumulative and long-term form of stigma that can undermine their sense of competence and social worth, whereas men encounter more immediate and recurring forms of stigma that are often managed through silence or bodily discipline. In both cases, the body becomes a public site of evaluation, reflecting how bodily stigma operates as an everyday social practice that contributes to the reproduction of gender inequality.

Finally, it is important to consider the broader social context in which this study was conducted. The data were collected during a period marked by increasing public discussion of the body, health, and weight management in Saudi society, particularly with the expansion of social media and image-based forms of comparison. This context may have heightened participants’ awareness of appearance-related expectations and intensified the anxieties related to body image and self-esteem described in their narratives.

Based on the principle of reflective research, the researchers acknowledges its social and cultural context and that this context may influence the choice of research topic and the interpretation and analysis of data. At the same time, this research stems from extensive academic engagement in the study of the body, aesthetic standards, and gender relations in Saudi society, in addition to research experience based on in-depth listening to individuals’ experiences in their daily contexts. This awareness of context is viewed as part of methodological rigor, not a constraint that diminishes the value of the analysis, as it contributes to enhancing transparency and critical reflection in the research process.

This study has several limitations that should be considered when interpreting the findings. First, the qualitative nature and relatively small sample size mean that the aim of the study was not statistical generalization but rather gaining an in-depth understanding of participants’ experiences. Therefore, the findings should be understood as reflecting the perspectives of the participants involved in this study rather than representing Saudi society as a whole. In addition, some participants were recruited through the researcher’s personal networks and snowball sampling. Although this approach is common in qualitative research, particularly when exploring sensitive topics, it may have introduced a degree of familiarity between the researcher and some participants, which could potentially influence how participants expressed their experiences. Furthermore, the reliance on individual interviews means that the results reflect participants’ perceptions at a specific point in time, and these perceptions may evolve as social or personal contexts change. Nevertheless, the diversity of participants’ ages and social backgrounds allowed the study to capture a range of experiences, helping to identify common patterns and gender differences in body image and self-esteem among the participants.

### Implications for practice

The findings of this study indicate that beauty standards in Saudi society are not merely individual preferences but rather function as social control mechanisms that influence body image and self-esteem in both women and men in distinct gendered ways. Therefore, any practical interventions aimed at promoting mental health and positive body image should extend beyond the individual and encompass the social contexts in which these standards are reproduced, such as the family, workplaces, educational institutions, and social media.

The results highlight the importance of developing media literacy programs that foster critical awareness of idealized images and visual practices prevalent on social media, particularly among young people, while considering gender differences in the nature of body image-related pressures. While body pressures for women are linked to social acceptance and appearance, for men they are linked to discourses of discipline, prestige, and competence.

The findings also suggest the need to redirect social discourse from focusing on physical appearance to its functions and mental health, thereby reducing constant self-monitoring and transforming the body from a site of moral judgment to one of self-care. The study also emphasizes the importance of raising awareness among families and employers about the cumulative impact of “spontaneous” or “joking” body language comments, as they play a role in promoting stigma and undermining social security and self-esteem.

### Limitations and future studies

Despite the contributions this study makes to understanding the relationship between beauty standards, body image, and self-esteem in the Saudi context, it is important to note several methodological limitations that simultaneously open avenues for future research.

First, the study relied on a qualitative approach based on individual interviews, which limits the statistical generalizability of the findings. However, this methodological constraint aligns with the study’s objective of achieving a deeper understanding of subjective experiences and social meanings associated with the body. This limitation highlights the importance of future studies employing quantitative or mixed methodologies to examine the broader prevalence of the patterns revealed in this study.

Second, the data were collected within a specific social and temporal context, characterized by a rise in public discourse surrounding the body, health, and appearance, particularly in light of the increasing influence of social media. Therefore, future comparative studies—whether across different time periods or among other Arab societies—may contribute to understanding how beauty standards have changed and their impact on body image and self-esteem across time and diverse cultural contexts.

Third, while the sample was diverse in terms of age and social background, it does not represent all segments of Saudi society, particularly in terms of regional or class diversity. This limitation opens the door for future research focusing on specific social groups, such as adolescents, the elderly, rural residents, or those working in particular professional sectors.

Fourth, the results indicate clear gender differences in body experiences and stigma, but the study did not delve deeply into other intersections such as social class, health status, or disability. Therefore, future studies could adopt an intersectional approach to gain a more comprehensive understanding of the complexities of body experiences in Saudi society.

In addition, it is also important to acknowledge that the gender of the interviewers may have influenced participants’ responses, particularly given the gender-sensitive nature of the topic and the cultural context of Saudi Arabia.

Finally, although individual interviews provided a safe space for expression, some participants—especially men—may have restricted their disclosure in accordance with prevailing gender expectations regarding emotional expression. This highlights the importance of employing diverse research methods in future studies, such as focus groups or gender-segregated interviews, to deepen our understanding of these experiences.

## Conclusion

This qualitative study sought to understand how body image and self-esteem are formed among women and men in Saudi society under prevailing beauty standards, by analyzing their daily experiences within various social contexts. The results showed that beauty standards function not merely as individual preferences, but as a social system that reproduces acceptance and exclusion through the body, directly and indirectly influencing self-esteem and social behavior.

The study reveals clear gender differences in the nature of pressures associated with body image. Women’s experiences are more closely linked to the logic of “conditional acceptance” and the constant monitoring of aesthetic details in family, professional, and social spaces, while men’s experiences tend to reframe aesthetic pressure within a discourse of discipline, prestige, and competence, with a notable presence of ridicule and casual comments as a form of daily stigma. In both cases, the body becomes a public space subject to evaluation and commentary, producing psychological and social effects that extend to symbolic withdrawal from certain situations, a reorganization of presence in social spaces, or the adoption of continuous self-monitoring practices.

This study contributes to the literature on body image and body stigma by highlighting the specificities of the Saudi context, where social transformations and the growing visual culture disseminated through social media intersect with traditional forms of social control. This interplay reinforces the production of standardized beauty criteria and deepens their impact on individuals. The findings underscore the need to address body image as a socio-cultural and psychological health issue, requiring interventions that extend beyond the individual level to encompass the family, school, workplace, and media discourse. This approach aims to foster more balanced and humane perceptions of the body and promote self-esteem that transcends the logic of appearance as a prerequisite for social acceptance.

## Data Availability

The raw data supporting the conclusions of this article will be made available by the authors, without undue reservation.
